# An inducible glycogen synthase-1 knockout halts but does not reverse Lafora disease progression in mice

**DOI:** 10.1074/jbc.RA120.015773

**Published:** 2020-12-10

**Authors:** Silvia Nitschke, Erin E. Chown, Xiaochu Zhao, Shoghig Gabrielian, Sara Petković, Dikran R. Guisso, Ami M. Perri, Peixiang Wang, Saija Ahonen, Felix Nitschke, Berge A. Minassian

**Affiliations:** 1 Division of Neurology, Department of Pediatrics, University of Texas Southwestern Medical Center, Dallas, Texas, USA; 2 Program in Genetics and Genome Biology, The Hospital for Sick Children Research Institute, Toronto, Ontario, Canada; 3 Department of Biochemistry, University of Texas Southwestern Medical Center, Dallas, Texas, USA

**Keywords:** Lafora disease (Lafora progressive myoclonic epilepsy, MELF), glycogen, glycogen synthase, glycogen storage disease, neuroinflammation, neurodegenerative disease, Lafora bodies, CAL, corpora amylacea–like, GFAP, glial fibrillary acidic protein, GYS1, glycogen synthase-1, IBA1, ionized calcium–binding adapter molecule 1, LB, Lafora body, LD, Lafora disease, PASD, periodic acid–Schiff–diastase, TAM, tamoxifen

## Abstract

Malstructured glycogen accumulates over time in Lafora disease (LD) and precipitates into Lafora bodies (LBs), leading to neurodegeneration and intractable fatal epilepsy. Constitutive reduction of glycogen synthase-1 (GYS1) activity prevents murine LD, but the effect of GYS1 reduction later in disease course is unknown. Our goal was to knock out *Gys1* in laforin (*Epm2a*)-deficient LD mice after disease onset to determine whether LD can be halted in midcourse, or even reversed. We generated *Epm2a*-deficient LD mice with tamoxifen-inducible Cre-mediated *Gys1* knockout. Tamoxifen was administered at 4 months and disease progression assessed at 12 months. We verified successful knockout at mRNA and protein levels using droplet digital PCR and Western blots. Glycogen determination and periodic acid–Schiff–diastase staining were used to analyze glycogen and LB accumulation. Immunohistochemistry using astrocytic (glial fibrillary acidic protein) and microglial (ionized calcium-binding adapter molecule 1) markers was performed to investigate neuroinflammation. In the disease-relevant organ, the brain, *Gys1* mRNA levels were reduced by 85% and GYS1 protein depleted. Glycogen accumulation was halted at the 4-month level, while LB formation and neuroinflammation were significantly, though incompletely, prevented. Skeletal muscle analysis confirmed that *Gys1* knockout inhibits glycogen and LB accumulation. However, tamoxifen-independent Cre recombination precluded determination of disease halting or reversal in this tissue. Our study shows that *Gys1* knockdown is a powerful means to prevent LD progression, but this approach did not reduce brain glycogen or LBs to levels below those at the time of intervention. These data suggest that endogenous mechanisms to clear brain LBs are absent or, possibly, compromised in laforin-deficient murine LD.

Lafora disease (LD) is teenage-onset progressive myoclonus epilepsy that is typically fatal within 10 years of symptom onset (1, 2). LD is caused by autosomal recessive mutations in either *EPM2A* or *NHLRC1* (also known as *EPM2B*), encoding laforin, a glucan phosphatase, and malin, an E3 ubiquitin ligase, respectively (3, 4, 5). The exact roles of both enzymes are unclear. It is, however, established that laforin and malin form a functional complex that regulates glycogen metabolism, with absence of either protein causing formation of malstructured glycogen (6, 7). LD is widely considered a glycogen storage disease (8, 9, 10, 11). The abnormal glycogen molecules precipitate and aggregate to form Lafora bodies (LBs), which continually accumulate, leading to symptom onset and then inexorably worsening neurodegeneration, seizures, and cognitive decline, culminating in vegetative state and death in status epilepticus and related complications (8).

Both laforin- and malin-deficient LD mouse models (12, 13, 14, 15) recapitulate the hallmarks of LD: glycogen accumulation, LB formation, and neuroinflammation (16, 17). In pursuit of a therapy for this intractable disease, one approach that emerged was to reduce glycogen synthase-1 (GYS1) activity, thereby interfering with chain elongation during glycogen synthesis, in order to prevent glycogen accumulation and LB formation. This approach proved successful with knockout of *Gys1* or *Ppp1r3c*, encoding a protein (also known as PTG) involved in GYS1 activation. Both gene knockouts prevented LB formation, neurodegeneration, and other features of the disease in LD mice (16, 17, 18, 19). These results established proof of principle that targeting *Gys1* for downregulation could be a promising therapeutic approach in LD. However, the mice in these studies had constitutively reduced GYS1. This left the question open as to how successful therapeutic targeting of *Gys1* could be when LBs are already present, *i.e.*, after disease onset and diagnosis.

We generated laforin-deficient (*Epm2a*^*−/−*^) mice with tamoxifen (TAM)-inducible *Gys1* knockout. We administered TAM at 4 months, after murine disease onset, and assessed subsequent disease progression at 12 months. We analyzed glycogen content, LB accumulation, and neuroinflammation to determine whether LD progression can be halted in midcourse, or even potentially reversed.

## Results

### Laforin-deficient conditional *Gys1* knockout mice

**Figure 1:**
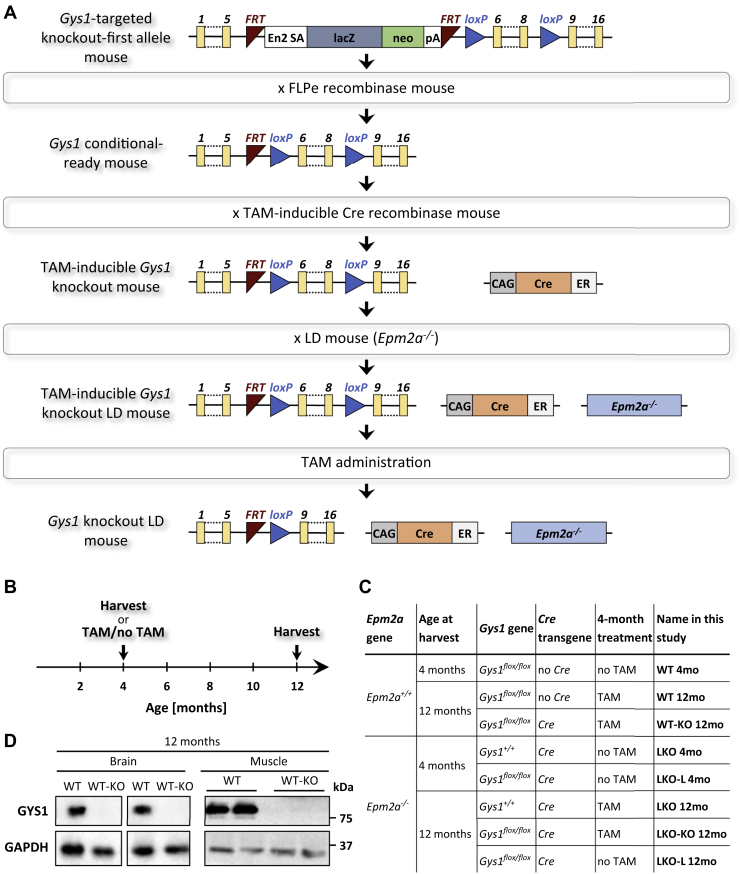
**Generation of Lafora disease (LD) model mice with tamoxifen (TAM)-inducible *Gys1* knockout.***A*, overview of breeding strategy. *B*, schematic of study timeline. *C*, overview of experimental groups of mice. LKO, laforin (*Epm2a*) knockout; WT, wild-type. *D*, western blots depicting brain and muscle GYS1 protein levels with GAPDH as the loading control. Duplicates are biological replicates. CAG, chicken beta actin promoter/enhancer coupled with the cytomegalovirus immediate-early enhancer; En2 SA, mouse engrailed 2 splice acceptor sequence; ER, mutant estrogen receptor 1 ligand-binding domain; FRT, flippase recombination target; lacZ, reporter gene encoding β-galactosidase; loxP, Cre recombinase recognition site; neo, neomycin resistance gene; pA, SV40 polyadenylation signal.

We generated conditional *Gys1* knockout mice to test whether genetic pharmacotherapy targeting *Gys1* following disease onset would lead to a halt or even reversal of LD progression. We used mice carrying a *Gys1*-targeting cassette with loxP sites flanking exons 6 to 8 together with TAM-inducible Cre to generate conditional *Gys1*-knockout mice in wild-type (WT) and *Epm2a*^*−/−*^ backgrounds (Fig. 1*A*). A subset of mice was harvested at 4 months, while the majority of mice were either TAM-treated or not treated and then aged until 12 months (Fig. 1*B*). Figure 1*C* gives an overview of all experimental mice, not only explaining their genotype and treatments but also introducing the names of each group used throughout the study. In addition to the WT and *Epm2a*^*−/−*^ control (WT, LKO) and *Gys1* knockout (WT-KO, LKO-KO) mice, we included LKO-L mice at 4 and 12 months. These LKO-L mice carried floxed *Gys1* and expressed Cre but were not TAM-treated. They served to evaluate whether TAM-independent Cre-mediated recombination (referred to as “Cre leakage”) occurred. At 12 months, WT-KO mice exhibited GYS1 protein knockdown in the brain and muscle (Fig. 1*D*), which clearly indicated that Cre-mediated *Gys1* recombination and knockout was successful.

### Tamoxifen-induced *Gys1* knockout halts glycogen and LB accumulation in the brain

**Figure 2:**
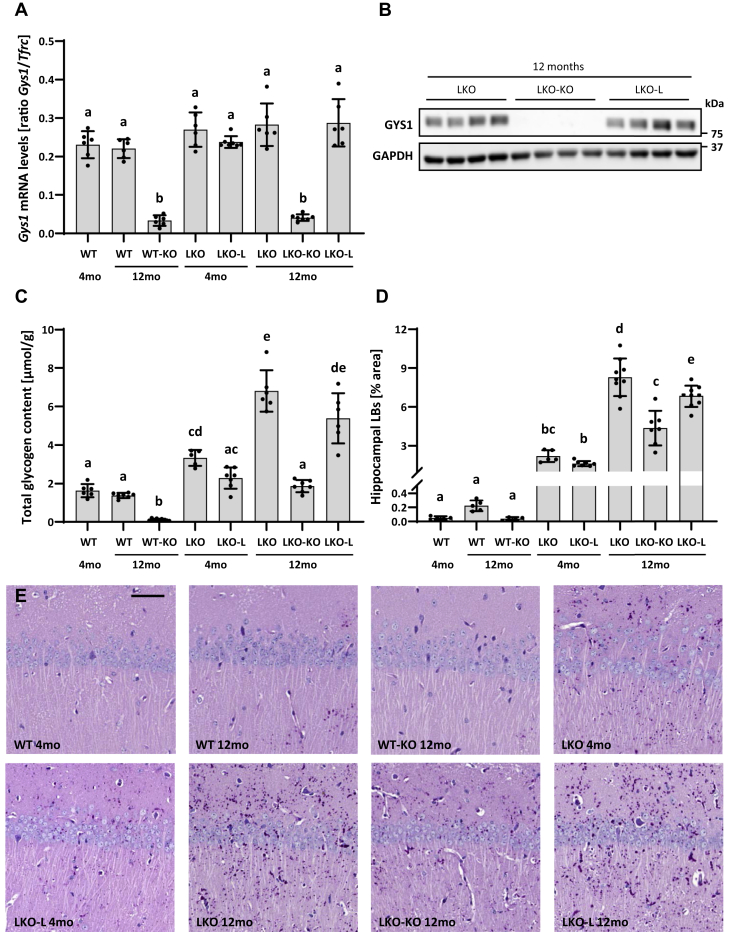
***Gys1* expression, glycogen, and Lafora body (LB) accumulation in the brain.***A*, *Gys1* mRNA levels in the brain. *Tfrc* served as the reference gene. *B*, western blots depicting brain GYS1 protein levels with GAPDH as the loading control. Replicate lanes are from individual animals. *C*, total glycogen content in the brain. *D*, LB quantification in the hippocampus. Error bars indicate SD. Statistical significance (*p* < 0.05) is denoted by different letters, while lack of significance is reflected by at least one shared letter. Where applicable, subsets of experimental groups underwent secondary follow-up statistical testing (Fig. S1). *E*, representative images of the PASD-stained hippocampus. Scale bar: 50 μm. PASD, periodic acid–Schiff–diastase.

To assess the effect of TAM treatment in the brain of conditional *Gys1*-knockout mice (WT-KO, LKO-KO) and potential Cre-leakage mice (LKO-L), we determined the frequency of recombination events by quantifying *Gys1* mRNA that still contained exons 6 to 8. Levels of intact *Gys1* mRNA were decreased by approximately 85% in both WT-KO and LKO-KO mice, while LKO-L mice showed no reduction and hence no Cre leakage at 4 or 12 months (Fig. 2*A*, Fig. S1*A*). Like WT-KO mice (Fig. 1*D*), LKO-KO mice exhibited strong knockdown of GYS1 protein levels, while no decrease in GYS1 was detected in LKO-L compared with LKO mice (Fig. 2*B*).

We then asked whether hallmarks of LD such as glycogen and LB accumulation in the brain were halted at the 4-month level, the time point of knockout induction, or perhaps even reversed. Glycogen determination confirmed an age-dependent increase in glycogen accumulation in LKO and LKO-L mice compared with WT in accordance with disease progression (Fig. 2*C*, Fig. S1*B*). There was a slight though not statistically significant trend toward less glycogen accumulation in LKO-L mice than in LKO control mice. Therefore, we directly compared LKO-KO mice with LKO-L mice in order to draw valid conclusions about disease halting or reversal. Doing so, we found clear stoppage in disease progression evidenced by LKO-L mice at 4 months having a very similar glycogen content as LKO-KO mice at 12 months (Fig. 2*C*, Fig. S1*B*). In WT-KO mice, glycogen levels were depleted, showing that *Gys1* expression was sufficiently reduced to almost completely prevent synthesis of normal soluble glycogen. A similar reduction in soluble glycogen is to be expected in LKO-KO mice, and therefore, it can be concluded that the glycogen detected in these mice is mainly insoluble glycogen.

Hippocampal LB quantification showed a small though significant amount of corpora amylacea–like (CAL) bodies (normal age-related astrocytic glucan inclusions (20, 21)) in 12-month-old WT mice and age-dependent LB accumulation in LKO and LKO-L mice (Fig. 2*D*, Fig. S1*C*). CAL body formation was prevented by *Gys1* knockout in WT-KO mice. LB accumulation was also significantly reduced in LKO-KO mice compared with LKO and LKO-L mice at 12 months, though slightly increased compared with LKO and LKO-L mice at 4 months. Therefore, *Gys1* knockout at 4 months did not completely halt but significantly slowed LB formation in the hippocampus. In LD mice, the density of LBs is highest in the hippocampus, a structure whose pathologies commonly underlie epileptogenesis (22). However, in order to show a more complete picture regarding the LB load in the whole brain, we also quantified LBs in two additional large brain regions, cerebellum and cortex (Fig. S2, *A*–*B*). The results are very similar to what we saw in the hippocampus with the exception that in the cerebellum, a complete halt, *versus* only slowing of LB accumulation, could be achieved in LKO-KO mice. Representative periodic acid–Schiff–diastase (PASD) images are shown for LB visualization in Figure 2*E* and Fig. S2*C*.

### Attenuated astrogliosis and microgliosis in the brain of conditional *Gys1*-knockout mice

**Figure 3:**
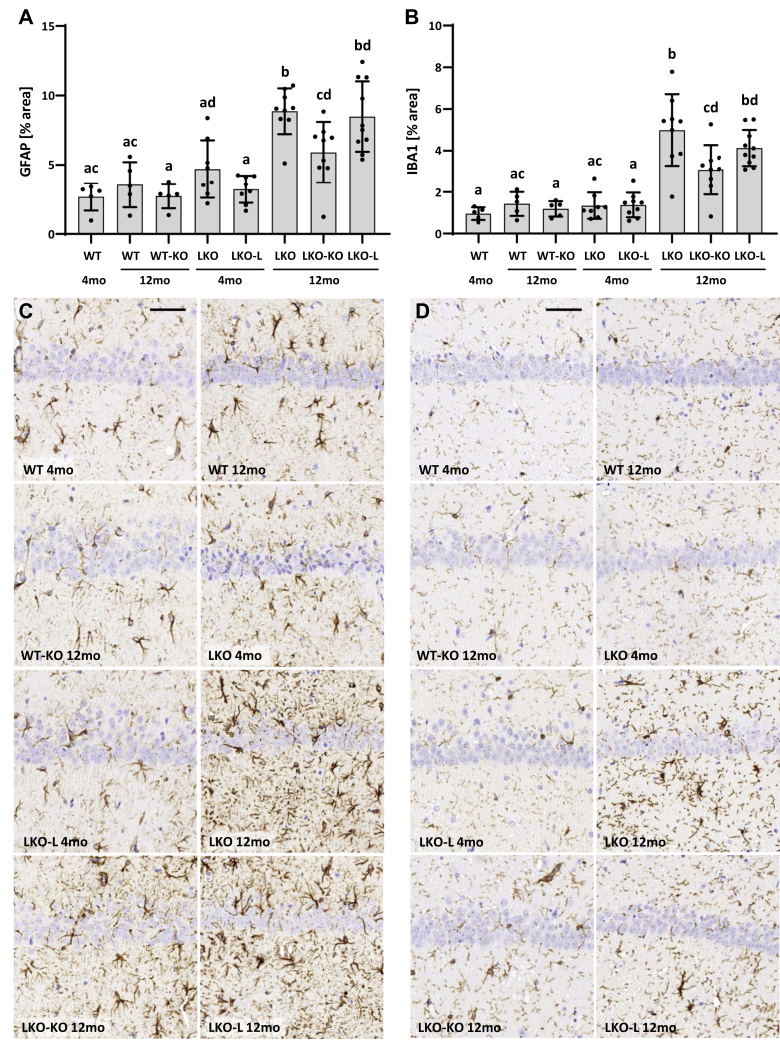
**Reactive gliosis in the brain.***A*–*B*, quantification of immunohistochemistry stain against GFAP (*A*) and IBA1 (*B*) in the hippocampus. Error bars indicate SD. Statistical significance (*p* < 0.05) is denoted by different letters, while lack of significance is reflected by at least one shared letter. Where applicable, subsets of experimental groups underwent secondary follow-up statistical testing (Fig. S3). C and D, representative images of GFAP (*C*) and IBA1 (*D*) immunohistochemistry in the hippocampus. Scale bars: 50 μm. GFAP, glial fibrillary acidic protein; IBA1, ionized calcium–binding adapter molecule 1.

Another hallmark of LD is glia-derived neuroinflammation (23). By performing immunohistochemistry for glial fibrillary acidic protein (GFAP) and ionized calcium–binding adapter molecule 1 (IBA1), we, respectively, assessed whether astrogliosis and/or microgliosis can be halted or reversed by targeting *Gys1*. In the hippocampus, WT and WT-KO mice did not exhibit any significant differences irrespective of *Gys1* genotype or age (Fig. 3, Fig. S3). LKO and LKO-L mice showed minimal (GFAP) or no (IBA1) neuroinflammation compared with WT mice at 4 months but significantly increased GFAP and IBA1 signals by 12 months, confirming increased hippocampal neuroinflammation with LD progression. Twelve-month-old LKO-KO mice showed partial rescue with GFAP and IBA1 signal levels between 4-month-old and 12-month-old LKO and LKO-L mice levels.

GFAP and IBA1 signals were also quantified in the cerebellum (Fig. S4). However, astrogliosis was not significantly increased in LKO or LKO-L mice at 12 months, while microgliosis was significantly increased but not rescued in LKO-KO mice. In the cerebellum, LD-associated neuroinflammation was not as pronounced as in the hippocampus and consequently the timeframe to determine a halt in progression of neuroinflammation was too short to observe significant rescue in LKO-KO mice.

### Prevention of the LD phenotype in the muscle

**Figure 4:**
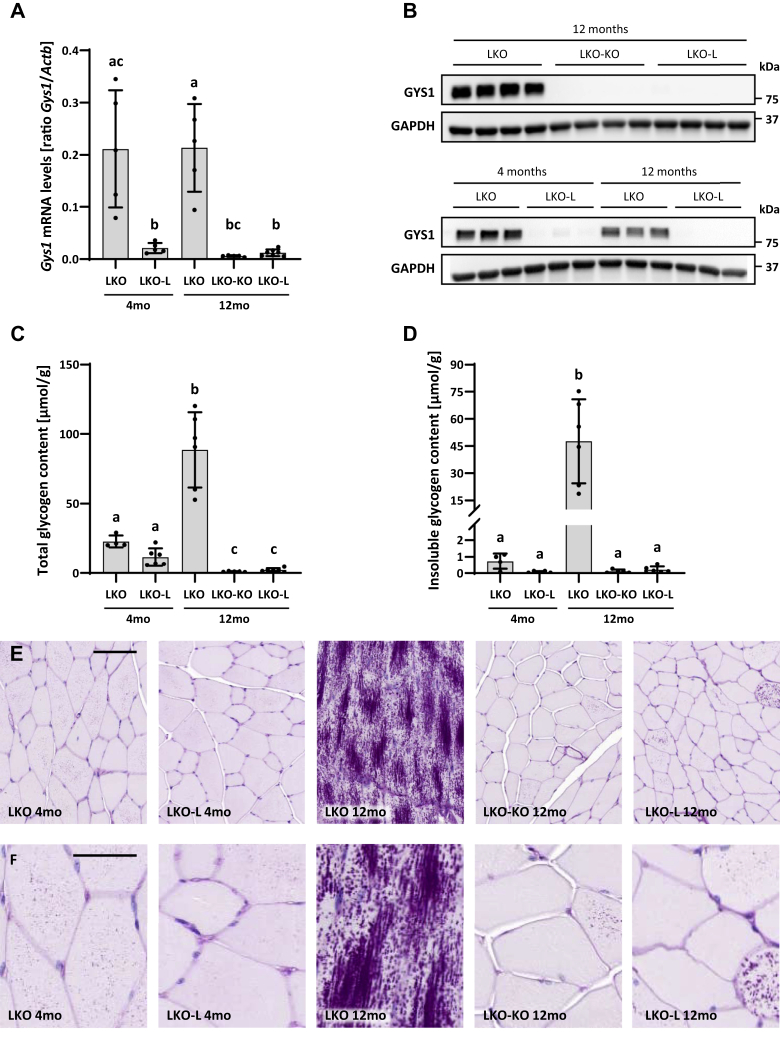
***Gys1* expression, glycogen, and Lafora body accumulation in the skeletal muscle.***A*, *Gys1* mRNA levels in the muscle. *Actb* served as the reference gene. *B*, western blots depicting muscle GYS1 protein levels with GAPDH as the loading control. Replicate lanes are from individual animals. *C*, total glycogen content in the muscle. *D*, insoluble glycogen content in the muscle. Error bars indicate SD. Statistical significance (*p* < 0.05) is denoted by different letters, while lack of significance is reflected by at least one shared letter. Where applicable, subsets of experimental groups underwent secondary follow-up statistical testing (Fig. S5). *E*–*F*, representative images of the PASD-stained muscle. Scale bars: 100 μm (*E*); 50 μm (*F*). PASD, periodic acid–Schiff–diastase.

While LD manifests as a neurological disease, LBs additionally accumulate in murine and patient skeletal muscle. We therefore analyzed the muscle to further assess the effect of *Gys1* knockout on glycogen and LB accumulation post disease onset. Like in the brain, we determined the extent of recombination events by quantifying the reduction of *Gys1* mRNA with exons 6 to 8 still present (Fig. 4*A*, Fig. S5*A*). Surprisingly, *Gys1* mRNA levels were already approximately 90% reduced in LKO-L mice at 4 months, indicating major Cre leakage in the muscle, which was not the case in the brain (Fig. 2*A*). At 12 months, *Gys1* mRNA levels were further reduced by 3.5-fold in LKO-KO and by approximately 2-fold in LKO-L mice, culminating in 97% and 94% knockdown, respectively, while LKO control mice remained unaffected. GYS1 protein levels in LKO-KO mice were as reduced as in WT-KO mice at 12 months (Fig. 1*E*, Fig. 4*B*). However, we detected a comparable depletion of GYS1 levels in LKO-L mice at 12 months. Even at 4 months, GYS1 levels were already reduced in LKO-L mice, confirming Cre leakage at the protein level in addition to that seen at the mRNA level.

Further analyses focused on the effect of TAM-induced and TAM-independent *Gys1* knockout on skeletal muscle total and insoluble glycogen content. For the latter, we used a recently established method that utilizes endogenously expressed metabolic enzymes to digest accessible soluble glycogen completely, leaving only the insoluble glycogen, which is not degradable (24). This method allowed us to determine whether changes in total glycogen content were mostly due to changes in soluble glycogen, insoluble glycogen, or both. LKO mice showed an increase in total and insoluble glycogen at 12 months, while the amount of insoluble glycogen was only approximately 3% of the total glycogen at 4 months (Fig. 4, *C*–*D*, Fig. S5, *B*–*C*). These results are in line with the extensive LB accumulation in LKO mice at 12 months as well as the comparatively small LB load at 4 months, with a substantial number of PASD-positive cells but very small bodies (Fig. 4, *E*–*F*). Already at 4 months, LKO-L mice exhibited 50% reduced levels of total glycogen and an almost complete depletion of insoluble glycogen, indicating that not only was disease progression attenuated but also levels of soluble glycogen were significantly reduced. Accordingly, fewer cells were PASD-positive with less/smaller LBs. The extent of LB formation in 12-month-old LKO-KO mice looked very similar to 4-month-old LKO-L mice, in accordance with the comparable insoluble glycogen content. However, in the LKO-KO mice, even the soluble glycogen was reduced further, as total glycogen was reduced by approximately 95% compared with 4-month-old LKO mice. We detected an approximately 2-fold higher amount of total and insoluble glycogen in LKO-L mice at 12 months than in LKO-KO mice. The slightly higher amount, though not statistically significant, is in line with the PASD results that show a few cells with relatively many LBs, which are probably cells where Cre leakage did not occur or only affected one of the two alleles. Taken together, our results show that *Gys1* knockout prevented the LD muscle phenotype; however, significant Cre leakage occluded assessment of disease reversal in this organ.

## Discussion

Hallmarks of LD are glycogen accumulation and formation of LBs, which are insoluble, glycogen-like particles, characterized by reduced branching and long chains (25). Therefore, reducing glycogen synthesis is an attractive avenue toward a therapy for the disease. From studying LD mouse models, we already know that reducing glycogen synthesis either by *Gys1* knockout or indirectly by knockout of PTG prevents LB formation, neurodegeneration, and other features of the disease such as impaired autophagy (16, 17, 18, 19). Potential strategies to target brain glycogen synthesis are multiple, including antisense oligonucleotides, other forms of RNA interference, genome editing, and small-molecule inhibitors (8). Targeting glycogen synthesis as a therapy for LD has the advantage of applicability to patients with mutations in either *EPM2A* or *NHLRC1*, as opposed to gene replacement therapies, which would require separate development for each subgroup. A second major advantage is that downregulating a function is comparatively more practicable at the present time than replacing a missing function across the entire brain. Conventional knockout mouse models showed that it is possible to prevent LB formation and other disease-associated features when the capability to synthesize glycogen is blocked with the animal still *in utero*. Here, we asked the clinically more relevant question, namely, what would the effect of the intervention be after disease onset.

We generated a conditional *Gys1*-knockout mouse, using TAM-induced Cre-mediated recombination, which enabled us to downregulate glycogen synthesis in laforin-deficient mice at 4 months when the LD neuropathological phenotype is already clearly detectable. We studied the hallmarks of LD, glycogen accumulation, LB formation, and consequent neuroinflammation. Interestingly, total brain glycogen in LKO-KO at 12 months was significantly lower than in LKO control mice at 4 months. Although we hoped to see a reversal, our results rather show a halt in disease progression for the following two reasons. Firstly, we expect the amount of soluble glycogen in LKO-KO mice to be reduced as strongly as in WT-KO mice, where the overall glycogen content was reduced by approximately 90%, because *Gys1* mRNA and protein levels suggest a similar extent of Cre recombination in WT-KO and LKO-KO mice. This indicates that we detected mostly insoluble glycogen in the *Gys1*-knockout mice (LKO-KO). Secondly, we need to take into consideration that the amount of total glycogen in 4-month-old LKO control mice is a combination of soluble and insoluble glycogen. In order to estimate the amount of insoluble (LB) glycogen at 4 months, we can subtract the amount of WT glycogen from the amount in LKO mice because we have shown that the amount of soluble glycogen is not altered in LD mice, at least in the muscle (26). This estimate gives approximately the same glycogen quantity as we see in LKO-KO mice at 12 months, further corroborating our conclusion of a halt in disease progression.

Strikingly, although still present, the halt in disease progression was not as complete when we look at the LB quantification results in the hippocampus. In the cerebellum, however, we observed a complete halt in LB formation which is more in line with our findings on total glycogen level, measured in whole brain samples. It could be that Cre recombination was slightly less efficient in the hippocampus than in other parts of the brain such as the cerebellum. The *Gys1* mRNA and protein levels are an average over the whole brain and do not reveal any potential disparities in recombination efficiency in different brain regions. An alternative explanation for differences seen in different parts of the brain could be cell type–specific differences in Cre recombination, being, for instance, less efficient in astrocytes than in other cell types (*e.g.*, neurons or other glial cells). It has been shown that a great number of LBs in LD mice are found in astrocytes (21, 27). Furthermore, it was found that the ratio between astrocytic and neuronal LBs can be different in different brain regions (21). Assuming this ratio is different in the hippocampus and cerebellum, cell type–specific differences in Cre recombination could explain the different extent of rescue seen in these two brain regions. Furthermore, glycogen metabolism in astrocytes is very different from glycogen metabolism in neurons (28, 29). These differences could result in different responses to reduced GYS1 levels. For example, GYS1 levels are much higher in astrocytes than in neurons, and it cannot be ruled out that the residual *Gys1* expression in astrocytes, if, for example, only one allele is knocked out, could still drive LB formation at a slow pace, while *Gys1* expression from only one allele in neurons may be too low to do so.

Neuroinflammation is a major feature of LD. As the disease progresses, reactive astrocytes and microglia appear and increase in number over time (16, 30, 31, 32), reflecting inflammatory and immune system responses. Intriguingly, 94% of the genes that are differentially expressed in both LD mouse models (*Epm2a*^*−/−*^ and *Nhlrc1*^*−/−*^) are related to inflammatory and immune system responses, further validating the latter as critical to LD pathophysiology (23). Our GFAP and IBA1 immunohistochemistry results show slower progression of neuroinflammation in the hippocampus of LKO-KO mice, in accordance with the hippocampal LB quantification results. In the cerebellum, where a complete halt in LB formation was observed, less pronounced neuroinflammation in 12-month-old LKO and LKO-L mice rendered it hard to draw conclusions regarding rescue of neuroinflammation in this brain region. However, there is a strong association between epilepsy and hippocampal pathology (reviewed in (22)) and our data from hippocampus confirm that the severity of neuroinflammatory and immune responses is tightly linked to disease progression, as previously shown (23, 30).

The main focus in this study was the brain as the clinically relevant organ in LD. However, to further characterize our conditional *Gys1* mouse model, we also analyzed skeletal muscle. Notably, we observed substantial TAM-independent Cre-mediated recombination in muscle, in contrast to the brain where *Gys1* mRNA and GYS1 protein levels did not indicate significant Cre leakage. The original publication of the Cre mice used in this study reported Cre leakage in a small number of sporadic cells (33), and this commonly used mouse line has since accrued almost 600 references on Mouse Genome Informatics. Many of these publications did not report inclusion of appropriate controls to enable assessment of Cre leakage. Welle *et al*. (34) did assess Cre leakage and similarly reported a leakage-induced 50 to 90% reduction in target mRNA in skeletal muscle by 4 months of age.

TAM-inducible Cre mice, such as the ones used in this work, express transgenic Cre recombinase fused to a mutant estrogen receptor ligand-binding domain. Cre–estrogen receptor fusion proteins are sequestered in the cytoplasm until exposure to 4-hydroxytamoxifen (active TAM metabolite) enables translocation to the nucleus. Despite this putative mechanism for temporal regulation of Cre recombination, Cre leakage is a well-documented phenomenon caused by incomplete trapping of Cre in the cytoplasm (35, 36). Experimental parameters including Cre expression level and duration (37, 38, 39) and target gene sensitivity to recombination (40, 41) have also been associated with Cre leakage likelihood. Accordingly, the strong Cre promoter (chicken beta actin promoter/enhancer and cytomegalovirus immediate-early enhancer) in our mice and the 4-month pre-TAM timeframe may have contributed to Cre leakage. In regard to tissue-specific leakage, it should be noted that variability in both TAM-induced and TAM-independent recombination across tissues and cell types has been reported with the Cre mice used here, despite ubiquitous Cre expression (42, 43). Tissue-specific differences in the local chromatin structure at the locus of the Cre transgene (random integration), as well as that of the target gene, could result in increased Cre expression (44, 45) and a higher loxP site accessibility (36, 40), respectively, in the muscle. While both factors could promote TAM-independent *Gys1* recombination, it cannot be ruled out that other unknown factors play a role. Overall, our muscle results provide further evidence that Cre leakage is a conserved and likely under-reported challenge of using TAM-inducible Cre regulation (45, 46). The difference in Cre leakage between the brain and muscle observed in this work highlights the importance of assessing Cre leakage in all tissues studied. Without including the LKO-L group, we would have overlooked Cre leakage in the muscle and our conclusions would have been very different—depicting reversal of LD in the muscle and conveying an incorrect clinically critical notion.

In the first months and even up to 3 years into the disease, patients with LD are neurologically still substantially preserved. Especially near onset, they are so healthy that they are nearly always misdiagnosed with the benign juvenile myoclonic epilepsy. As such, an approach that drastically slows or blocks disease progression, as we have shown in the present work in the *Epm2a*^*−/−*^ mouse model of the disease, will be highly desirable. In the present era, patients with LD are diagnosed comparatively swiftly because *EPM2A* and *NHLRC1* are included in most epilepsy gene panels, and therefore, most new patients would benefit. This study also aimed at uncovering whether downregulating glycogen synthesis could result in removal of existing LBs. Perhaps mechanisms such as autophagy no longer overwhelmed with constant LB formation could clear LBs accumulated to that point. Since we did not observe a net reduction in LBs despite GYS1 depletion, robust LB clearance mechanisms in the brain seem to be absent, at least in the laforin-deficient mouse model.

Several treatment approaches for LD have been suggested, and many are currently being probed in preclinical studies (8, 11). Some of these approaches aim at reducing GYS1 levels or activity. Those include administration of antisense oligonucleotides that promote degradation of *Gys1* mRNA or of small-molecule inhibitors of GYS1. A recent publication describes the successful development of small-molecule inhibitors of GYS1 and demonstrates substantial inhibition of GYS1 in cell lysates (10). While *in vivo* efficacy remains to be proven, these strategies may eventually present viable treatment options for LD. However, if our results in mice translate to human LD, these GYS1-directed therapies may at most prevent further progression, but not reverse the disease. Therefore, GYS1-targeting therapies may have to be administered early in the disease progression with potentially limited benefits in patients with more advanced disease states. For the latter, other approaches in development, such as delivery of LB-digesting amylase to the brain (9), would be needed. There, the question will be to what degree the accompanying neuropathology will reverse following LB clearance.

## Conclusions

Our results clearly show that slowing or halting LD progression after LBs have already formed is possible in the LD mouse model. Unfortunately, clearance of existing LBs was not observed. Our findings if translated to patients emphasize the importance of early diagnosis in treatments targeting glycogen synthesis downregulation.

## Experimental procedures

### Mice

Mice were housed with environmental enrichment in individually ventilated cages at 20 to 22 °C and had access to water and food *ad libitum*. Animals were sacrificed at 4 or 12 months of age by cervical dislocation. The brain and hind-limb muscle were harvested with half of each tissue immediately frozen in liquid nitrogen followed by −80 °C storage and the other half fixed in 10% buffered formalin followed by paraffin embedding. For brain analyses, both sexes were used with no indication of a disease phenotype–related sexual dimorphism, while only male mice were used for muscle analyses. Animal procedures were approved by The Centre for Phenogenomics Animal Care Committee and in compliance with the Canadian Council for Animal Care Guidelines.

### Generation of laforin-deficient conditional *Gys1*-knockout mice

Mouse embryonic stem cells (C57BL/6N strain) containing a *Gys1*-targeted knockout-first allele with conditional potential (*Gys1*^*tm1a(EUCOMM)Wtsi*^; (47)) were obtained from the European Mouse Mutant Cell Repository. The *Gys1*^*tm1a(EUCOMM)Wtsi*^ allele carries an FRT-flanked lacZ reporter and neomycin resistance sequence in intron 5 and loxP sites flanking exons 6 to 8 (Fig. 1*A*). Targeted embryonic stem cells were aggregated with morula and implanted in pseudo-pregnant females. Chimeric mice were crossed with B6N-*Tyr*^*c-Brd*^/BrdCrCrl mice to test for germline transmission. F1 mice were crossed with FLPe mice (B6;Cg-Tg(ACTFLPe)9205Dym/J; JAX stock #005703; (48)) to excise the FRT-flanked gene-trap cassette, yielding mice with a conditional-ready *Gys1*^*tm1c*^ allele.

These *Gys1*^*tm1c*^ mice were crossed with TAM-inducible Cre mice (B6.Cg-Tg(CAG-cre/Esr1∗)5Amc/J, JAX stock #004682; (33)). Upon TAM induction, Cre excises exons 6 to 8, leading to a premature termination codon after amino acid 293 (*Gys1*^*tm1d*^ allele; full-length GYS1: 738 amino acids), which has been shown to be effective in knocking out *Gys1* (49).

To assess therapeutic efficacy of *Gys1* knockout after LD onset, mice with conditional-ready *Gys1* and TAM-inducible Cre were crossed with laforin-deficient LD model mice (*Epm2a*^*−/−*^; (12)). See Table 1 for genotyping primers.

**Table 1:** Genotyping primers

Primer name	Purpose	Sequence [5’-3’]	Amplicon size [bp]
GeneTrap-F	Detection of gene trap cassette in knockout-first with conditional potential *Gys1*^*tm1a*^ allele	AGGGTCAAGTAGCGGTGTTG	207
GeneTrap-R		GTGGGAAAGGGTTCGAAGTT	
LoxP-F	Detection of loxP site in conditional-ready *Gys1*^*tm1c*^ allele	AGGGTCAAGTAGCGGTGTTG	270 (*Gys1*^*+*^), 458 (*Gys1*^*flox*^)
LoxP-R		TCAACTTCACAGGCAAAAACC	
Cre-F	Detection of *Cre* transgene	GCGGTCTGGCAGTAAAAACTATC	102
Cre-R		GTGAAACAGCATTGCTGTCACTT	
Epm2a-F	Detection of both *Epm2a*^*+*^ and *Epm2a*^*-*^ alleles	GCATCGGCTGTAAGTTAGCC	620 (*Emp2a*^*+*^), 430 (*Epm2a*^*-*^)
Epm2a-WT-R	Detection of *Epm2a*^*+*^ allele	CGTGTGTCCATTCTCCAGAA	
Epm2a-KO-R	Detection of *Epm2a*^*-*^ allele	AGCGTATTCAATAACCCTTAAT	

### Tamoxifen administration

TAM administration *via* intraperitoneal injection was determined as a reliable way to knock down *Gys1*. For intraperitoneal injections, 20 mg/ml of TAM-free base (Sigma, #T5648) was dissolved in corn oil (Sigma, #C8267) by rotating in a light-blocking tube overnight at 37 °C. Mice received three 0.225-mg/g injections evenly spaced across 5 to 8 days.

### *Gys1* expression analysis

RNA was extracted using the Qiagen RNeasy Lipid Tissue Mini Kit for the brain and RNeasy Mini Kit for the muscle (#74804, #74104). Extraction was performed according to the manufacturer’s instructions, using a 1-cc syringe and 21-g needle for tissue homogenization. DNase I digestion was performed to eliminate genomic DNA (DNase I Amplification Grade, Invitrogen, #18068-015 or DNase I, RNase-free, Thermo Scientific, #EN0521), followed by cDNA synthesis with SuperScript First-Strand Synthesis System (Invitrogen, #11904-01), using 1.45 μg of brain RNA and 0.73 μg of muscle RNA for this final step with a total volume of 20 μl.

*Gys1* expression analysis was performed using the Bio-Rad QX200 Droplet Digital PCR (ddPCR) System. Transferrin receptor (*Tfrc*) was used as a reference gene in brain samples and actin beta (*Actb*) in muscle samples. For *Gys1* and *Tfrc*, custom TaqMan assays were used (Gys1-F: CAGAGCAAAGCACGAATCCA, Gys1-probe: TTATGGGCACCTGGAC, Gys1-R: CATAGCGGCCAGCGATAAAG; Tfrc-F: TGCCTAATATACCTGTGCAAACAATC, Tfrc-probe: CAAGAGCTGCTGCAGAA, Tfrc-R: TTCCTTCCATTTTTCCAAATAGCT), while for *Actb*, a predesigned one (Mm01205647_g1, #4331182), all from Thermo Fisher Scientific. The *Gys1* primer/probe set binds in exons 6 and 7 which are absent in *Gys1*-knockout mice. The 20-μl reaction mix consisted of 10 μl of 2× ddPCR SuperMix for Probes (Bio-Rad, #186-3023), 1 μl of *Gys1* assay (FAM-labeled), 1 μl of reference gene assay (VIC-labeled), 0.5 μl (muscle) or 3 μl (brain) of cDNA, and 7.5 μl or 5 μl of nuclease-free water. Cycling conditions were 95 °C for 10 min, followed by 45 cycles of 94 °C for 30 s and 60 °C for 1 min, and eventually 98 °C for 10 min on a Life Technologies Veriti thermal cycler. Data were analyzed using QuantaSoft v1.4 (Bio-Rad). *Gys1* was analyzed in duplex reaction mode together with the respective reference gene, and the results were expressed as the ratio of *Gys1* to the reference gene. No-template controls were run in parallel with study samples.

### Protein analysis

Lysate preparation and immunodetection of GYS1 and GAPDH was conducted as previously described (26).

### Glycogen determination

Glycogen extraction and digestion using amyloglucosidase as well as glucose determination were performed as previously described (25). For analysis of insoluble muscle glycogen, ground tissue was incubated at 37 °C for 2.5 h prior to glycogen extraction in KOH (24).

### Histological analysis

Paraffin-embedded brain or muscle tissue was sectioned and stained using PASD or immunohistochemical protocols at The Centre for Phenogenomics Pathology Services, Toronto. For immunohistochemistry, GFAP and IBA1 were used as neuroinflammation markers. Antibodies were diluted 1:2000 (rat anti-GFAP, Invitrogen, #130300) or 1:1500 (rabbit anti-IBA1, Wako, #01919741). Citrate buffer at pH 6 was used for antigen retrieval. Stained slides were scanned using a Pannoramic digital slide scanner (20× objective; 3DHistech), and representative images taken. LBs as well as GFAP and IBA1 signals were quantified in different parts of the brain using a method established in the image analysis program HistoQuant (3DHistech) by defining LBs or GFAP/IBA1 signals based on pixel color. Values are expressed as % area.

### Statistics

Mice used in this study were assigned to one of eight experimental groups that differed in three dimensions: laforin genotype (WT or *Epm2a*^*−/−*^), age at harvest (4 or 12 months), and *Gys1* genotype, which depended on the presence of *Gys1* loxP sites, Cre transgene, and TAM administration (Fig. 1*D*). Quantitative data are represented as group averages with standard deviation (SD). All statistics were performed with GraphPad Prism 8.4.2. To allow for an appropriate statistical comparison of all eight groups, initial statistical analyses of dependent variables (*e.g.*, brain *Gys1* mRNA level, brain glycogen level) were performed by Welch’s one-way ANOVA (overall eight groups) followed by post hoc tests with Dunnett T3 multiple comparison correction. Statistically significant differences between means are assumed for *p* < 0.05. In main text figures, letters were used to denote if means were statistically different or not. Groups that have no letter in common are significantly different, while those that share at least one letter are statistically not different. In addition, where applicable, subsets of experimental groups underwent secondary follow-up testing. When analyzing effects of two specific dimensions at a time on dependent variables, two-way ANOVA was performed followed by post hoc tests with Bonferroni’s multiple comparison correction of *p* values. Figs. S1–S5 display means of groups compared in these secondary tests as well as significance levels as indicated. In cases where post hoc significance levels between two groups differ in the initial one-way ANOVA (overall eight groups) and the secondary ANOVAs (selected groups), statistical significance (letter code) in the main figures is in accordance with the secondary test.

## Data availability

All data described are either presented in the article or are in the supporting information.

## Conflict of interest

The authors declare that they have no conflicts of interest with the contents of this article.
