# Supplementary material for: An inducible glycogen synthase-1 knockout halts but does not reverse Lafora disease progression in mice
Source: J Biol Chem. 2020 Dec 10;296:100150. doi: 10.1074/jbc.RA120.015773 (PMC7857511; doi:10.1074/jbc.RA120.015773)
Supplement: Supplementary Figures S1–S5 [file mmc1.pdf]

An inducible glycogen synthase-1 knockout halts but does not reverse Lafora disease progression in mice

Silvia Nitschke Ph.D.[1,2,4], Erin E. Chown M.Sc.[2,4], Xiaochu Zhao B.Sc.[2], Shoghig Gabrielian B.Sc.[2], Dikran R. Guisso[1], Sara Petković B.Sc.[2], Ami M. Perri M.Sc.[2], Peixiang Wang Ph.D.[2], Saija J. Ahonen Ph.D.[2], Felix Nitschke Ph.D.[1,3,5], Berge A. Minassian M.D.[1,2,5]

[1]Division of Neurology, Department of Pediatrics, University of Texas Southwestern Medical Center, Dallas, TX 75390, USA
[2]Program in Genetics and Genome Biology, The Hospital for Sick Children Research Institute, Toronto, ON M5G 0A4, Canada
[3]Department of Biochemistry, University of Texas Southwestern Medical Center, Dallas, TX 75390, USA
[4]These authors contributed equally
[5]Co-corresponding authors

Supporting information – material included:

Fig. S1
Fig. S2
Fig. S3
Fig. S4
Fig. S5

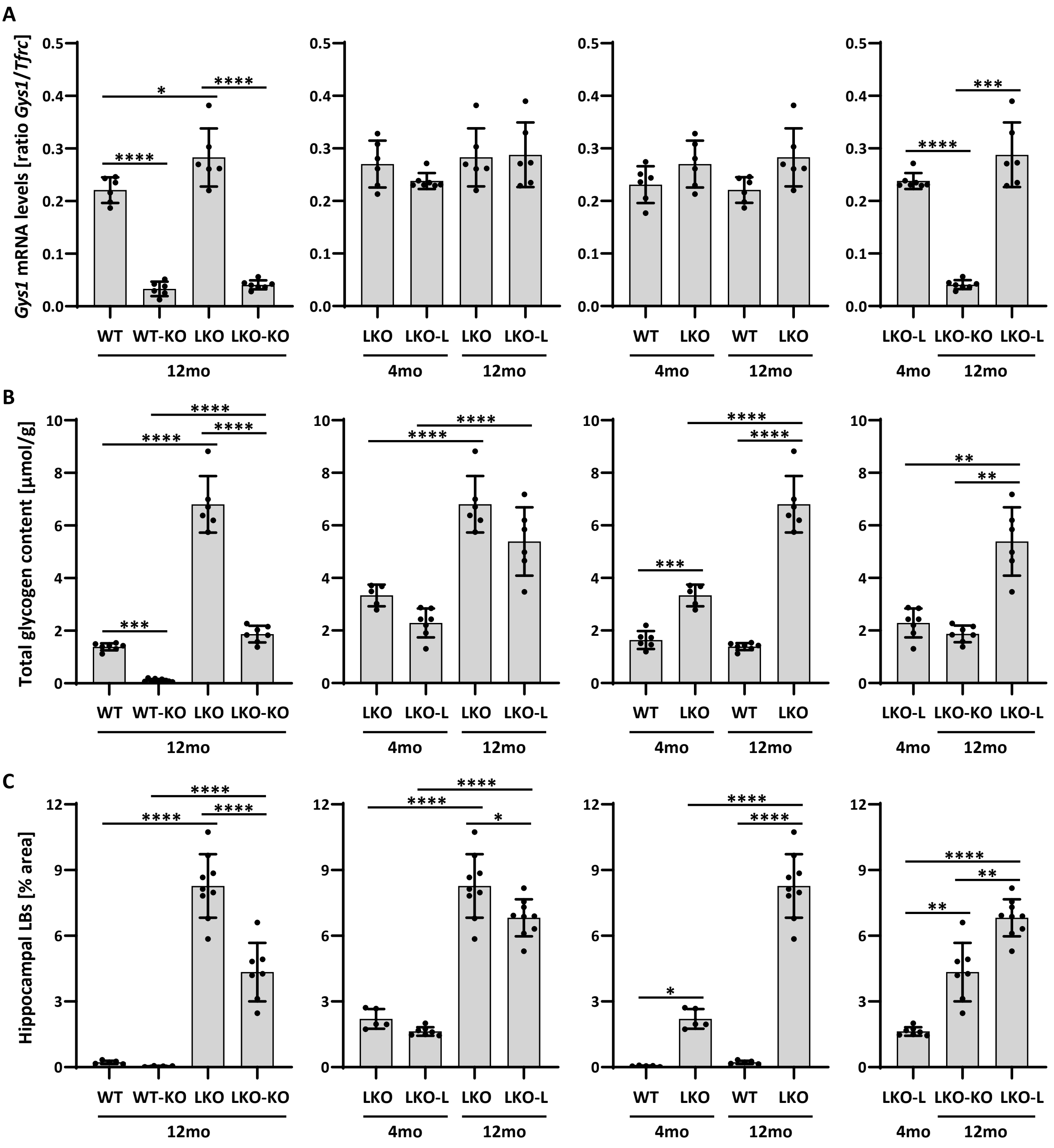

Figure S1. Follow-up statistical testing for *Gys1* mRNA levels (A), total glycogen content (B), and Lafora body (LB) quantification (C) in the brain. Two-way ANOVA for *Gys1* and *Epm2a* genotype (column 1), potential tamoxifen (TAM)-independent Cre recombination (Cre leakage) and age (column 2), and *Epm2a* genotype and age (column 3) as well as Welch's one-way ANOVA for TAM effect in isogenic mice (column 4). Error bars indicate SD. $p < 0.05$ *, $p < 0.01$ **, $p < 0.001$ ***, $p < 0.0001$ ****. Corresponding figure in the main text is Fig. 2.

A

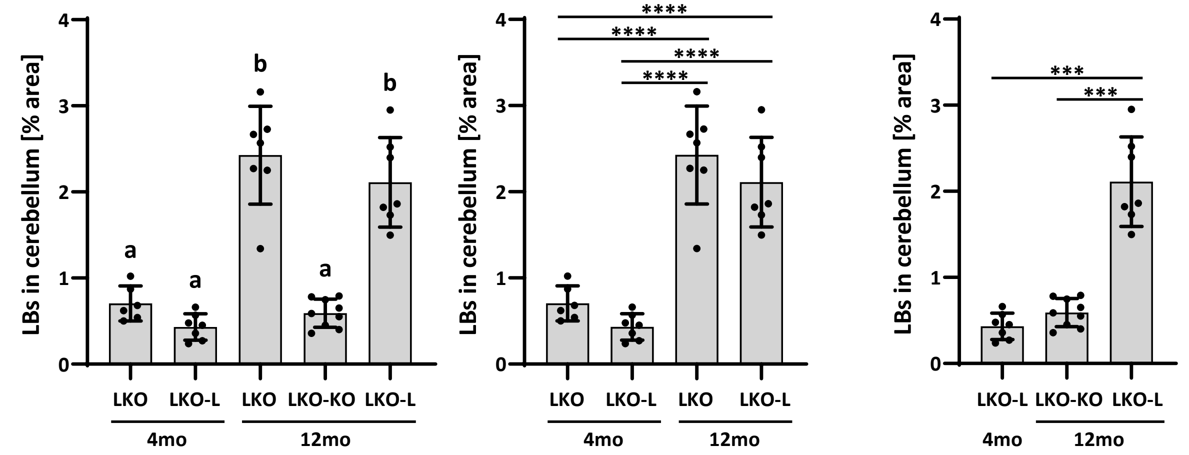
LBs in cerebellum [% area]
4
3
2
1
0
a
a
b
a
b
LKO
LKO-L
LKO
LKO-KO
LKO-L
4mo
12mo
****
****
****
****
LKO
LKO-L
LKO
LKO-L
4mo
12mo
***
***
LKO-L
LKO-KO
LKO-L
4mo
12mo

B

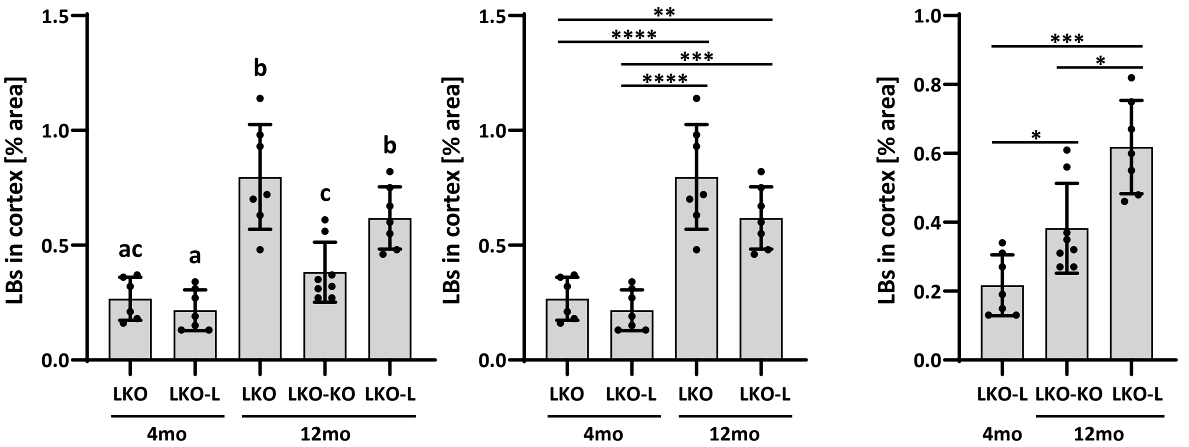
LBs in cortex [% area]
1.5
1.0
0.5
0.0
ac
a
b
c
b
LKO
LKO-L
LKO
LKO-KO
LKO-L
4mo
12mo
**
****
***
****
LKO
LKO-L
LKO
LKO-L
4mo
12mo
1.0
0.8
0.6
0.4
0.2
0.0
***
*
*
LKO-L
LKO-KO
LKO-L
4mo
12mo

C

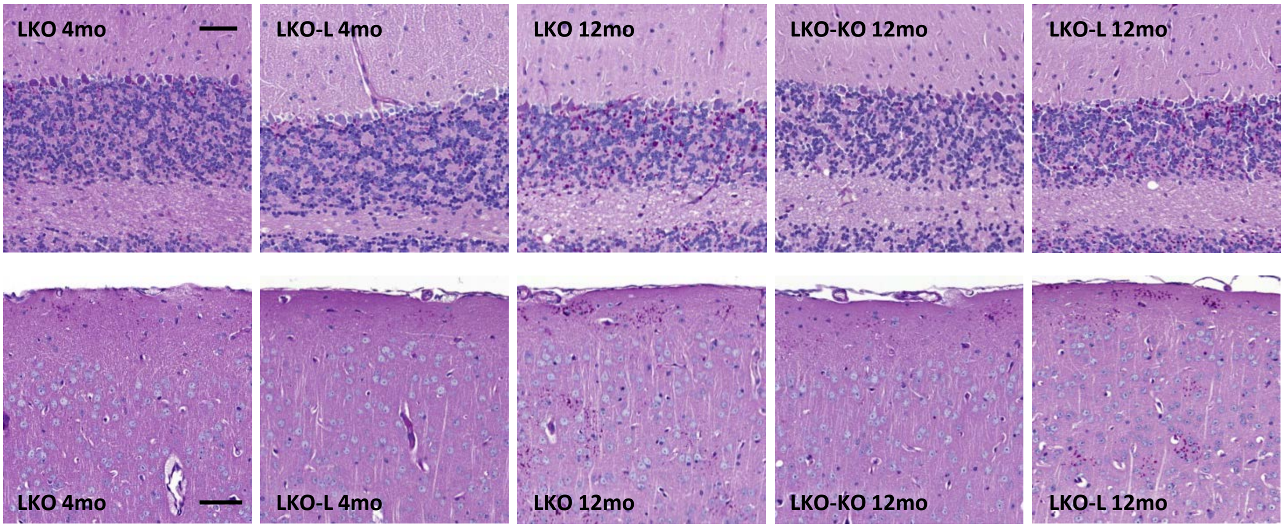
LKO 4mo
LKO-L 4mo
LKO 12mo
LKO-KO 12mo
LKO-L 12mo
LKO 4mo
LKO-L 4mo
LKO 12mo
LKO-KO 12mo
LKO-L 12mo

Figure S2. Lafora body quantification in cerebellum (A) and cortex (B). Error bars indicate SD. Statistical significance ($p < 0.05$) is denoted by different letters (column 1, Welch's one-way ANOVA), while lack of significance is reflected by at least one shared letter. Where applicable, subsets of experimental groups underwent secondary follow-up statistical testing, using two-way ANOVA for potential tamoxifen (TAM)-independent Cre recombination (Cre leakage) and age (column 2) and Welch's one-way ANOVA for TAM effect in isogenic mice (column 3). $p < 0.05$ *, $p < 0.01$ **, $p < 0.001$ ***, $p < 0.0001$ ****. C, Representative images of PASD stained cerebellum (top) and cortex (bottom). Scale bar: 50 µm.

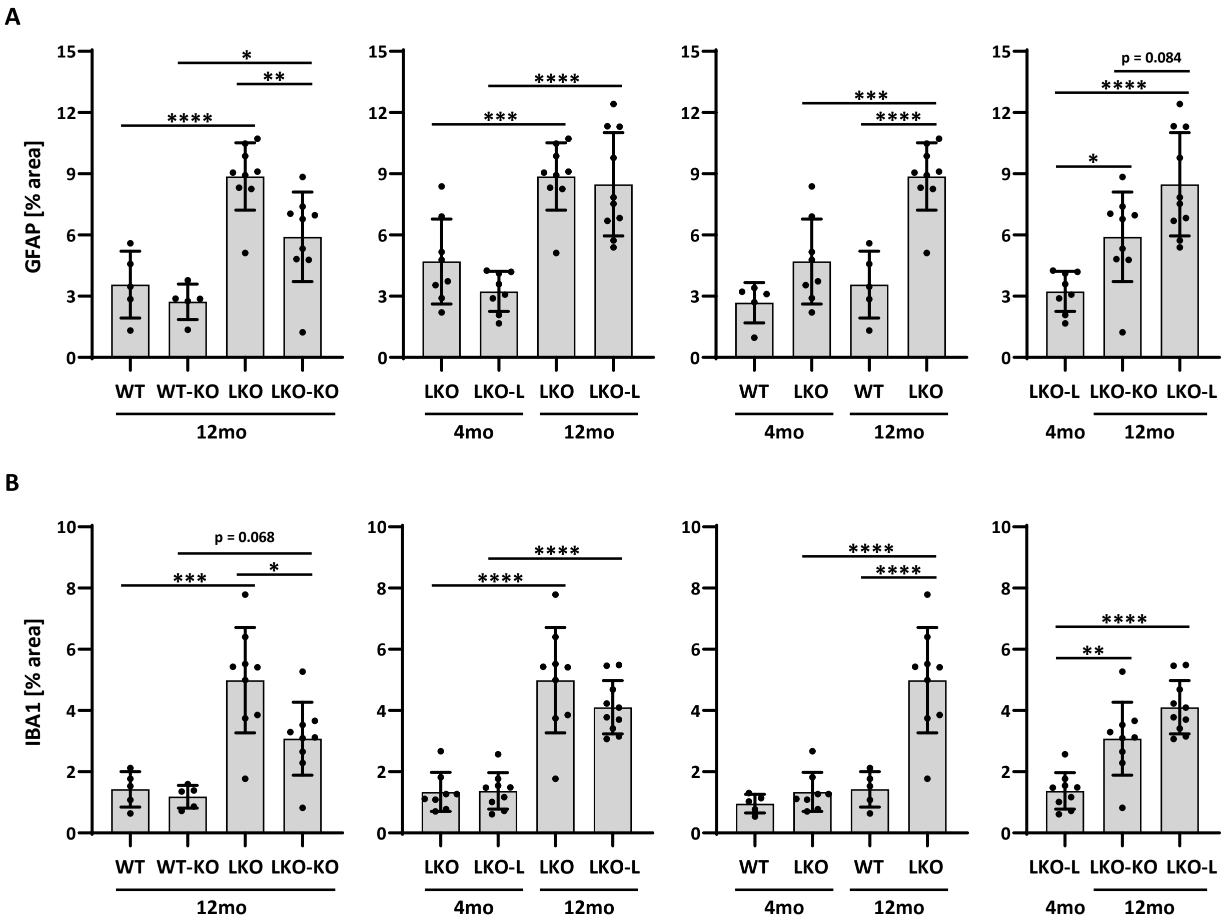

Figure S3. Follow-up statistical testing for GFAP (A) and IBA1 (B) immunohistochemical analysis in the hippocampus. Two-way ANOVA for *Gys1* and *Epm2a* genotype (column 1), potential tamoxifen (TAM)-independent Cre recombination (Cre leakage) and age (column 2), and *Epm2a* genotype status and age (column 3) as well as Welch's one-way ANOVA for TAM effect in isogenic mice (column 4). Error bars indicate SD. $p < 0.05$ *, $p < 0.01$ **, $p < 0.001$ ***, $p < 0.0001$ ****. Corresponding figure in the main text is Fig. 3.

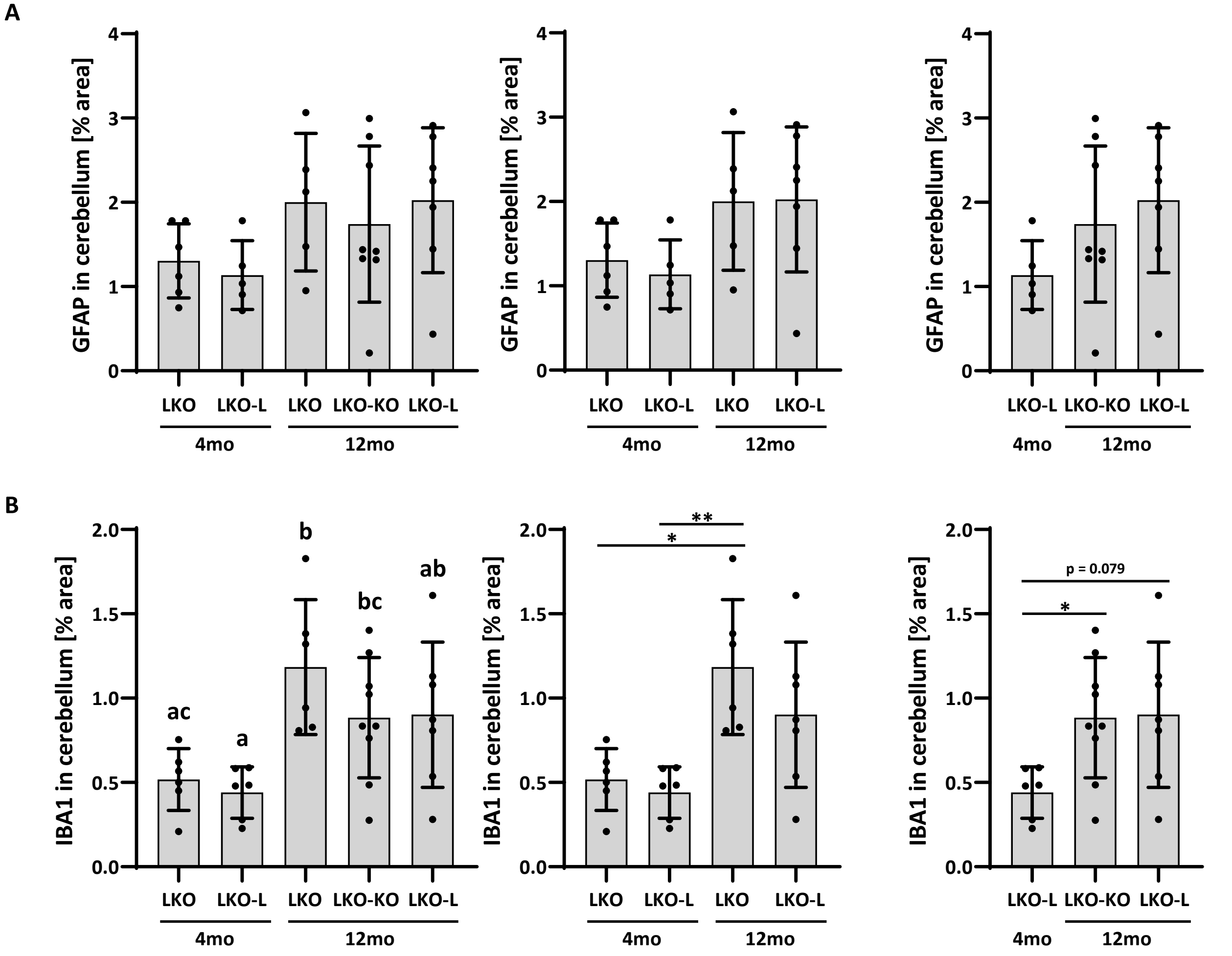

Figure S4. Quantification of GFAP (A) and IBA1 (B) immunohistochemical stain in cerebellum. Error bars indicate SD. Statistical significance ($p < 0.05$) is denoted by different letters (column 1, Welch's one-way ANOVA), while lack of significance is reflected by at least one shared letter. Where applicable, subsets of experimental groups underwent secondary follow-up statistical testing, using two-way ANOVA for potential tamoxifen (TAM)-independent Cre recombination (Cre leakage) and age (column 2) and Welch's one-way ANOVA for TAM effect in isogenic mice (column 3). $p < 0.05$ *, $p < 0.01$ **.

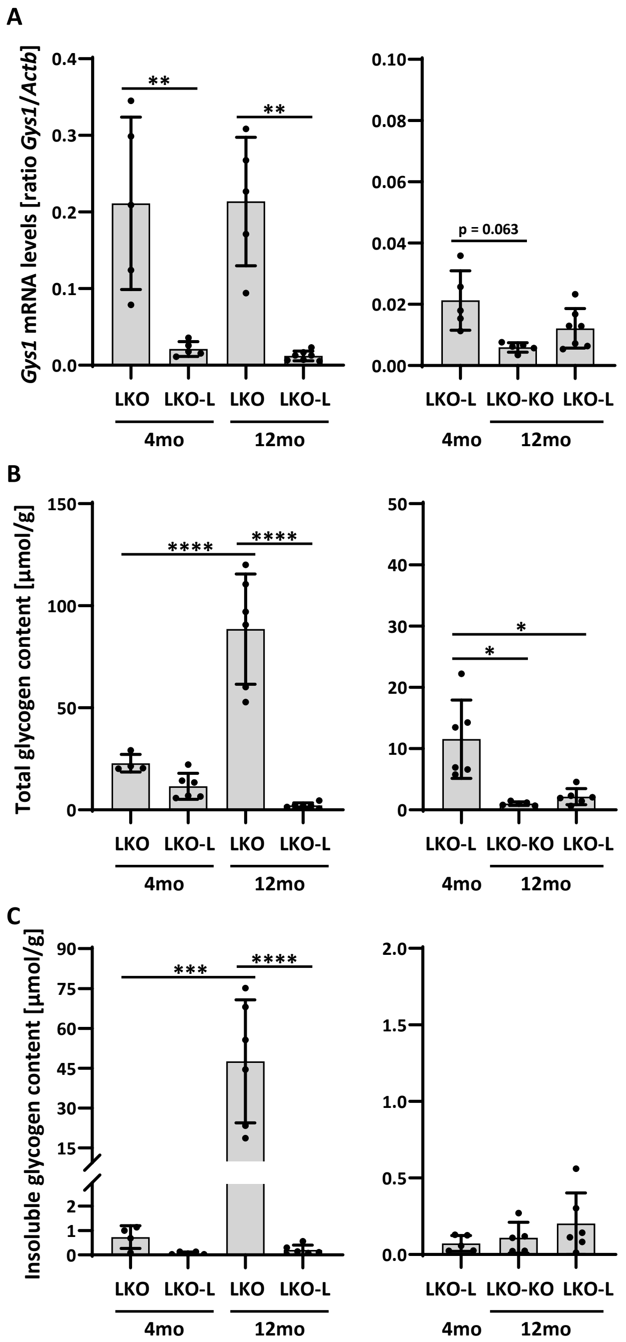

Figure S5. Follow-up statistical testing for *Gys1* mRNA levels (A), total glycogen content (B), and insoluble glycogen analyses (C) in the muscle. Two-way ANOVA for potential tamoxifen (TAM)-independent Cre recombination (Cre leakage) and age (column 1) and Welch's one-way ANOVA for TAM effect in isogenic mice (column 2). Error bars indicate SD. $p < 0.05$ *, $p < 0.01$ **, $p < 0.001$ ***, $p < 0.0001$ ****. Corresponding figure in the main text is Fig. 4.
